# Viral suppressors of the RIG-I-mediated interferon response are pre-packaged in influenza virions

**DOI:** 10.1038/ncomms6645

**Published:** 2014-12-09

**Authors:** Swantje Liedmann, Eike R. Hrincius, Cliff Guy, Darisuren Anhlan, Rüdiger Dierkes, Robert Carter, Gang Wu, Peter Staeheli, Douglas R. Green, Thorsten Wolff, Jonathan A. McCullers, Stephan Ludwig, Christina Ehrhardt

**Affiliations:** 1 Institute of Molecular Virology (IMV), Center for Molecular Biology of Inflammation (ZMBE), University of Muenster, Von-Esmarch-Street 56, D-48149 Muenster, Germany; 2 Department of Infectious Diseases, St. Jude Children’s Research Hospital, 262 Danny Thomas Place, Memphis, Tennessee 38105-3678, USA; 3 Department of Immunology, St. Jude Children’s Research Hospital, 262 Danny Thomas Place, Memphis, Tennessee 38105-3678, USA; 4 Department of Computational Biology, St. Jude Children’s Research Hospital, 262 Danny Thomas Place, Memphis, Tennessee 38105-3678, USA; 5 Institute of Virology, University Medical Center Freiburg, Hermann-Herder-Street 11, D-79104 Freiburg, Germany; 6 Division of Influenza and Other Respiratory Viruses, Seestraβe 10, Robert Koch-Institut, D-13353 Berlin, Germany; 7 Department of Pediatrics, University of Tennessee Health Sciences Center, 50 N. Dunlap, Memphis, Tennessee 38103, USA; 8 Cluster of Excellence Cells in Motion, University of Muenster, Muenster, Germany

## Abstract

The type I interferon (IFN) response represents the first line of defence to invading pathogens. Internalized viral ribonucleoproteins (vRNPs) of negative-strand RNA viruses induce an early IFN response by interacting with retinoic acid inducible gene I (RIG-I) and its recruitment to mitochondria. Here we employ three-dimensional stochastic optical reconstruction microscopy (STORM) to visualize incoming influenza A virus (IAV) vRNPs as helical-like structures associated with mitochondria. Unexpectedly, an early IFN induction in response to vRNPs is not detected. A distinct amino-acid motif in the viral polymerases, PB1/PA, suppresses early IFN induction. Mutation of this motif leads to reduced pathogenicity *in vivo*, whereas restoration increases it. Evolutionary dynamics in these sequences suggest that completion of the motif, combined with viral reassortment can contribute to pandemic risks. In summary, inhibition of the immediate anti-viral response is ‘pre-packaged’ in IAV in the sequences of vRNP-associated polymerase proteins.

## Abstract (web summary)

It is unclear how incoming influenza viruses counteract the cells’ first line of defence, the interferon (IFN) response. Here Liedmann *et al.* show that a distinct amino-acid motif in polymerases PB1 and PA, which are packaged in the viral particles, inhibit early IFN induction.

## Graphical abstract

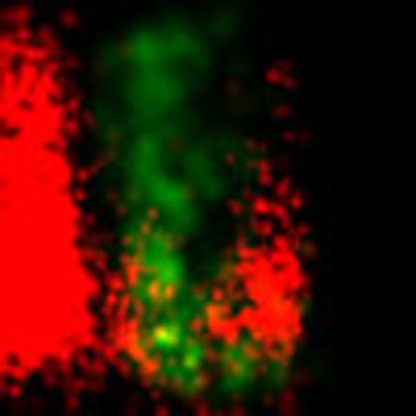

The type I interferon (IFN) system is a central host defence mechanism that recognizes invading pathogens and rapidly establishes an anti-microbial state to restrict viral and bacterial infections. To ensure efficient replication, pathogens have faced an evolutionary challenge to develop strategies to circumvent, resist or counteract activation of the type I IFN response.

During negative-strand RNA virus infection, the RNA helicase retinoic acid inducible gene I (RIG-I) detects viral ribonucleoprotein complexes (vRNPs) as one pathogen-associated molecular pattern among others by combined recognition of RNA secondary base-pairing structures and 5′-triphospate termini^1^,^2^. Following detection of vRNPs, RIG-I interacts with TRIM25 and is recruited to mitochondria by the scaffold protein MAVS, finally leading to the activation of the TBK-1/IRF-3 IFNβ induction pathway^3^,^4^. Previous studies of this process have focused on the role of newly synthesized vRNPs accumulating in the cell hours after initial infection. One recent report, focusing on the bunyaviruses Rift Valley fever virus and La Crosse virus, suggested that incoming, encapsidated RNA virus genomes in the early stages of infection may induce the type I IFN response independent of replication^2^. In contrast, very recently it was reported that induction of type I IFN by influenza viruses requires viral RNA synthesis and nuclear export. Incoming vRNPs were potentially ruled out as predominant inducer^5^.

Taking into account that viruses must attenuate the type I IFN response to establish efficient replication, the question arises if and how RNA viruses control early recognition of internalized vRNPs. For influenza A viruses (IAVs), the most prominent type I IFN antagonist is the nonstructural protein 1 (NS1). NS1 suppresses the type I IFN response by interacting with RIG-I^6^,^7^ and TRIM25 (ref. 8). Very recently, first evidence arose that NS1 is maybe present in the virion^9^. Overall functionality of incorporated NS1 and the impact on the type I IFN response still need to be explored, but as the number of NS1 proteins per virion is very low (mean=4.2, s.d.=3.2), it might not be sufficient for inhibition of the early type I IFN response potentially activated by internalized vRNPs^9^. This would imply the existence of an additional NS1-independent mechanism of type I IFN antagonism specifically targeting the earliest type I IFN response.

Here we visualize IAV vRNPs in three-dimensional, high-resolution light microscopy as helical-like structures associated with the mitochondrial outer membrane. We identify the polymerase proteins PB1 and PA as part of the type I IFN inhibitory strategy evolved by IAV and suggest a specific amino-acid motif to interact with RIG-I and subsequently inhibit RIG-I-mediated type I IFN signalling. We suggest PB1/PA-mediated type I IFN inhibition to be targeted towards the early induction of type I IFN as PB1 and PA are already present when internalized vRNPs are sensed by RIG-I and are located in close proximity to the site of RIG-I activation.

## Results and Discussion

### STORM revealed vRNPs as helical structures at mitochondria

**Figure 1:**
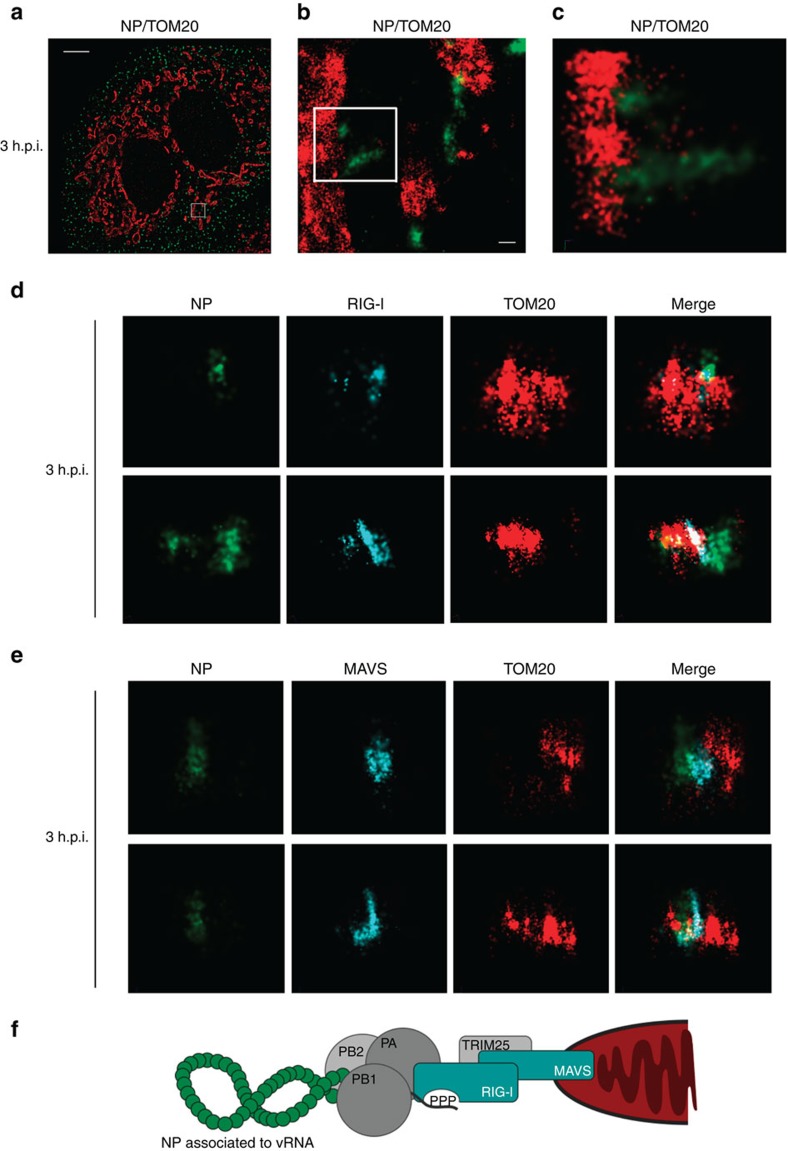
High-resolution visualization of internalized IAV vRNPs revealed helical-like structure in close proximity to RIG-I and MAVS at the mitochondrial membrane. (**a**–**e**) A549 cells were infected with H1N1-WT (multiplicity of infection (MOI)=50) and prepared for immunofluorescence analysis 3 h post infection (h.p.i.). (**a**–**c**) Three-dimensional stochastic optical reconstruction microscopy (STORM) revealed internalized vRNPs (green: NP-AF568) as helical-like structure (Supplementary Movie 1) in close association with the outer mitochondrial membrane (red: TOM20-AF647). (**a**) Wide shot, scale bar represents 5 μm, (**b**) zoom in, scale bar represents 200 nm and (**c**) zoom in 3D render. (**d**,**e**) vRNPs (green: NP-atto488) are in close proximity to (**d**) RIG-I (cyan: RIG-I-AF568) and (**e**) MAVS (cyan: MAVS-AF568) at the outer mitochondrial membrane (red: TOM20-biotin-streptavidin-AF647). Shown are representative images from three independent experiments each with at least 40 analysed cells. (**f**) Model of a mitochondria-associated vRNP. Coloured shapes indicate structure visualized in STORM analysis.

Using spinning disk laser scanning confocal microscopy, we visualized vRNPs localized in the mitochondrial network at 3 h post infection (h.p.i.). At this time point, vRNPs were not imported into the nucleus of human lung epithelial cells yet, whereas nuclear import was completed at 5 h.p.i. (Supplementary Fig. 1a). Therefore, we chose the 3 h.p.i. time point to investigate initial immune responses independent of viral replication in a physiological infection situation. In three-dimensional stochastic optical reconstruction microscopy (STORM), internalized vRNPs appeared as elongated, spiral structures (helical-like) of about 100–200 nm in length associated with the mitochondrial outer membrane (Fig. 1a–e, Supplementary Fig. 1b–f and Supplementary Movie 1), supporting previous structural determination of helical vRNPs by cryo-EM^10^,^11^. At the same time RIG-I and the type I IFN signalling integrator MAVS are localized to the outer surface of the mitochondria and are distinctly positioned next to the vRNPs (Fig. 1d,e), suggesting trafficking of RIG-I-bound vRNPs to the mitochondria followed by interaction with MAVS (hypothesized model shown in Fig. 1f).

Although the apparent close proximity of either RIG-I or MAVS with vRNPs would suggest activation of the type I IFN-inducing pathway, we were not able to detect induction of the pathway at this time point (Supplementary Fig. 2). This emphasized the idea that before the expression of IAV genes, proteins of the vRNPs must inhibit RIG-I-mediated type I IFN induction.

### Incoming vRNPs prevent type I IFN induction via PB1/PA

**Figure 2:**
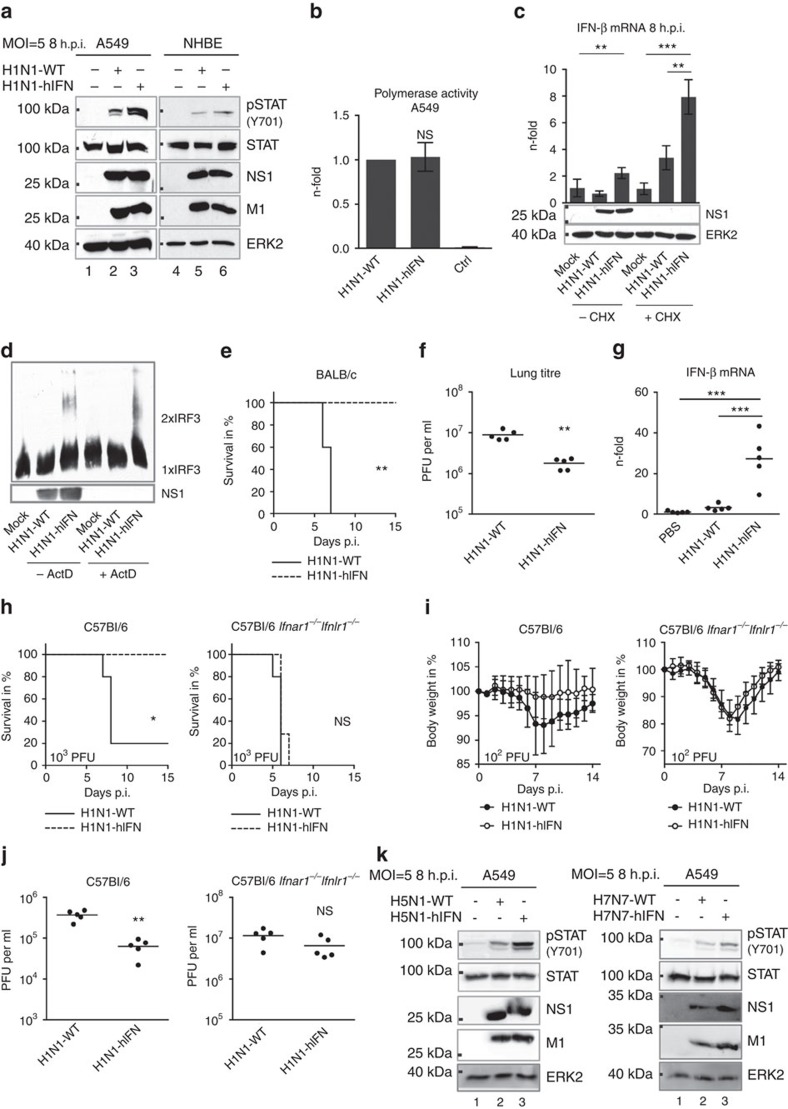
The ESIE-motif within PB1 and PA mediates type I IFN response. (**a**) A549 cells or NHBEs infected with the indicated viruses (multiplicity of infection (MOI)=5) were prepared for WB analysis 8 h.p.i. Enhanced type I IFN response following disruption of the ESIE-motif was analysed (pSTAT1 Y701). (**b**) Polymerase activity was measured in A549 cells. Cells not expressing PB1 served as control (Ctrl). (**c**) A549 cells treated with cycloheximide (CHX) or left untreated were infected with the indicated viruses (MOI=5). Elevated *IFNβ*-mRNA levels were measured following disruption of the ESIE-motif 8 h.p.i. independent of CHX treatment; CHX treatment was controlled by WB analysis of NS1. (**d**) A549 cells treated with actinomycin D (ActD) or left untreated were infected with the indicated viruses (MOI=5). Formation of IRF3 oligomers was measured following the disruption of the ESIE-motif 8 h.p.i. independent of ActD treatment; ActD treatment was controlled by WB analysis of NS1. (**e**–**j**) Mice (*n*=5 per group) were infected i.n. with 10^2.5^ plaque-forming units (PFU) (BALB/c) and 10^2^ PFU or 10^3^ PFU (C57Bl/6 and *Ifnar1*^*−/−*^*Ifnlr1*^*−/−*^). Mice were examined daily for (**e**,**h**) survival and (**i**) body weight loss for 14–15 days p.i. (**f**,**j**) Virus lung titres were determined 3 days p.i. (**g**) Elevated mRNA levels of *IFNβ* were measured 2 days p.i. Depicted are fold changes compared with PBS-inoculated mice. (**k**) A549 cells were infected with the indicated viruses (MOI=5) and prepared for WB analysis 8 h.p.i. Conservation of type I IFN regulatory properties of the ESIE-signature among IAV subtypes was analysed (pSTAT1 Y701) upon infection with highly pathogenic H5N1 and H7N7 viruses. (**a**,**d**,**k**) Shown are representative results from three independent experiments. (**b**,**c**) Bars show mean±s.d. of three independent experiments. (**f**,**g**,**j**) Each dot represents an individual mouse; bars show mean. For statistical analysis, (**b**,**f**,**j**) Student’s *t*-test, (**c**,**g**) analysis of variance with Tukey’s test, (**e**,**h**) log-rank (*χ*^2^) test for statistical analysis of Kaplan–Meier survival data and (**i**) Student’s *t*-test analysis (Holm–Sidak method) were performed; *P<0.05, **P<0.01, ***P<0.001, NS=not statistically significant. Full western blots are shown in Supplementary Fig. 4a–f.

We identified an amino-acid signature in the polymerase proteins PB1 (398E/524S/563I) and PA (351E)—referred to as ESIE-motif—that determines type I IFN inhibitory properties, as previously observed^12^. Disruption of the motif resulted in an enhanced type I IFN response upon viral infection as indicated by a stronger phosphorylation of STAT1 in human lung and bronchial epithelia (Fig. 2a, Supplementary Figs 3a,b, 4a,b) and elevated mRNA levels of IFN-related genes (Supplementary Fig. 3c) independent of any detectable changes in polymerase activity (Fig. 2b). Elevated mRNA levels of IFN-β were observed even in the presence of the translational elongation inhibitor cycloheximide that prevents viral protein synthesis and thereby viral replication (Fig. 2c, Supplementary Fig. 4c). Also, analysis of the oligomeric state of IRF3 revealed activation of the protein even upon blocking primary viral transcription with actinomycin D, confirming internalized vRNPs as one stimulus of the RIG-I-mediated type I IFN response (Fig. 2d, Supplementary Fig. 4d). Differences to a recent study by Killip and co-workers that seemingly ruled out internalized vRNPs as inducer of the type I IFN response^5^ are likely due to the type I IFN antagonistic properties of the polymerase proteins expressed by the analysed virus strains. The respective PB1 and PA proteins harbour amino acids in the ESIE-motif expected to antagonize IFN induction sufficiently, which might explain the lack in an early type I IFN response.

### The IFN antagonism of PB1/PA enhances virulence *in vivo*

To explore the functionality of the ESIE-motif in the context of complex immune regulatory processes within an organism, we infected mice and monitored survival, viral lung load and expression of IFN-β in mouse lungs. A mouse strain-independent reduction in morbidity and mortality was accompanied by diminished viral lung titres and elevated IFN-β mRNA levels attributable to abrogation of the domain (Fig. 2e–j, Supplementary Fig. 3d,e), suggesting a central immune regulatory function of the ESIE-motif for viral fitness. The functional link between the type I IFN antagonistic function of the ESIE motif and virulence was verified in mice lacking functional receptors for both type I and type III IFN (*Ifnar1*^*−/−*^*Ifnlr1*^*−/−*^). In such mice, pathogenicity and replication behaviour of wild-type and mutant virus were indistinguishable (Fig. 2h–j). Comparable results were seen in *Stat1*^*−/−*^ mice (data not shown). The type I IFN antagonistic function of the ESIE-motif is independent of the IAV subtype, as also in highly pathogenic H5N1 and H7N7 viruses the complete disruption of the motif caused an enhanced type I IFN response (Fig. 2k, Supplementary Fig. 3f,g and 4e,f).

### PB1/PA-mediated IFN antagonism complements the action of NS1

We next generated a recombinant NS1 mutant virus (H1N1-NS1 96/97) lacking the ability to inhibit the type I IFN response via interaction with TRIM25 (ref. 8). Disruption of either the TRIM25-mediated type I IFN antagonistic properties of NS1 or of the ESIE-motif within PB1/PA separately caused a similar reduction in mortality and viral lung load, whereas the combination of both alterations had an additive effect (Supplementary Fig. 5a,b). This argues for an autonomous IFN antagonistic function of PB1/PA with a contribution to viral fitness similar to that of NS1-mediated TRIM25 inhibition. Further, the putative temporal differences in utility of the two systems suggest complementary functions in inhibition of the early and late activities of type I IFN.

### Recruitment of vRNPs to mitochondria is affected by PB1/PA

**Figure 3:**
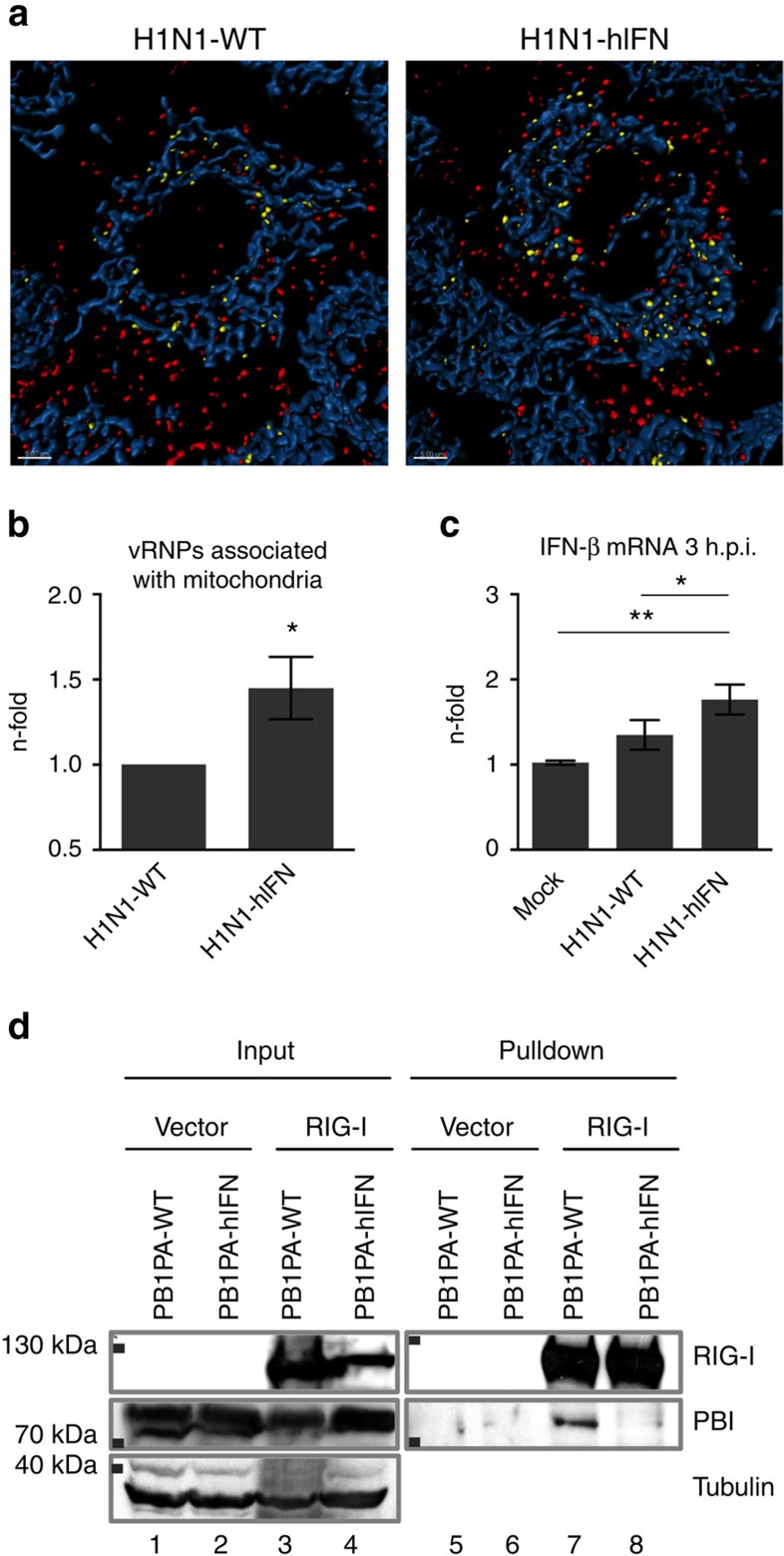
Disruption of the ESIE-motif results in enhanced recruitment of incoming vRNPs to mitochondria. (**a**) A549 cells were infected with the indicated viruses (multiplicity of infection (MOI)=50) and prepared for immunofluorescence analysis 3 h.p.i. Distance of vRNPs (<0.5 μm yellow, >0.5 μm red: NP-AF568) to mitochondria (blue: TOM20-AF647) was determined using spinning disk laser scanning confocal microscopy in at least 40 cells per group. Scale bars represent 5 μm. Shown are representative images from three independent experiments; quantification is presented in **b**. (**b**) Numbers of vRNPs associated to mitochondria (<0.5 μm) were normalized to the total number of vRNPs detected. Percentages of H1N1-hIFN vRNPs are depicted in relation to H1N1-WT vRNPs (n-fold). (**c**) A549 cells were infected with the indicated viruses (MOI=5) and prepared for qRT–PCR-analysis. Elevated mRNA levels of *IFNβ* were measured following disruption of the ESIE-motif 3 h.p.i.; bars show mean±s.d. of three independent experiments. (**d**) A549 cells were co-transfected with pcAGGS-RIG-I-FLAG or empty vector and pcDNA3-H1N1-PB1-WT/ pcDNA3-H1N1-PA-WT or pcDNA3-H1N1-PB1-hIFN/pcDNA3-H1N1-PA-hIFN and prepared for immunoprecipitation. Complex formation of RIG-I and PB1/PA according to a functional ESIE-motif was analysed by co-immunoprecipitation followed by probing for RIG-I and PB1 (the original blot is shown in Supplementary Fig. 4g). Shown are representative results from three independent experiments. For statistically analysis, (**b**) Student’s *t*-test and (**c**) analysis of variance with Tukey’s test for multiple comparisons were performed *P<0.05, **P<0.01.

To further analyse the mechanism of disruption of the type I IFN pathway, we used spinning disk laser scanning confocal microscopy to visualize internalized vRNPs and analysed the distance of vRNPs to mitochondria. Numbers of vRNPs associated to mitochondria were normalized to total numbers of vRNPs. We found that disruption of the ESIE-motif caused enhanced recruitment (1.5-fold) of incoming vRNPs to mitochondria (Fig. 3a,b) resulting in elevated IFN-β mRNA levels (1.5-fold) 3 h.p.i. (Fig. 3c). This indicates a spatial-temporal interplay of vRNP-RIG-I recruitment to mitochondria and type I IFN induction. The interaction with mitochondria-associated MAVS is a consequence of RIG-I activation as early step in the type I IFN signalling pathway. These results suggest that the type I IFN antagonistic properties of PB1/PA are targeted especially to the early induction phase via internalized vRNPs. The polymerase proteins bind to the 5′end of the viral genome, suggesting close proximity to interacting RIG-I. Co-immunoprecipitation (IP) of overexpressed proteins revealed complex formation of RIG-I and PB1/PA, indicating a direct interaction of these proteins, independent of the basal interaction of RIG-I with vRNPs mediated by the RNA 5′-triphosphate termini. This interaction was dependent on a functional ESIE-motif (Fig. 3d, Supplementary Fig. 4g). Based on these data, we suggest interaction of PB1/PA with RIG-I as potential mechanism to interfere with RIG-recruitment to mitochondria.

Insufficient structural data are available to determine the precise positions of the amino-acid residues forming the ESIE-motif in the secondary structure. A suggested model of the IAV polymerase heterotrimer predicts the E351 of PA to be on the protein surface and in proximity to PB1 (ref. 13). Based on this, we suggest the ESIE-motif as functional motif formed through the tertiary structure of the influenza polymerase heterotrimer that directly interacts with RIG-I and subsequently inhibits RIG-I-mediated type I IFN signalling, completing the picture of the type I IFN inhibitory capacity of the polymerase proteins^12^,^13^,^14^,^15^,^16^. PB1 and PA are already present when internalized vRNPs are sensed by RIG-I and are located in close proximity to the site of RIG-I activation. First evidence for the presents of NS1 in influenza virions^9^ give rise to the assumption that co-evolution of several type I IFN counteracting strategies has taken place.

### Amino-acid 398 in PB1 is evolutionary dynamic

Phylogenetic analysis to explore the occurrence of the ESIE-motif in IAV lineages revealed evolutionary variability of the position 398 in the PB1 protein, whereas the other three amino acids of the motif were highly conserved. Multiple swine and avian IAV lineages with a sustained D to E change at position 398 in the PB1 protein suggest selection of a fully intact ESIE-motif (Supplementary Fig. 6a,b). Focusing on swine IAV lineages, a D to E change occurred in the North America ‘classical’ swine lineage in the mid-1970s and persists in circulating strains until today (Supplementary Fig. 7). The 2009 pandemic H1N1 virus derived its genes from a reassortment event of the triple reassortant swine H1N2 lineage and the Eurasian swine H1N1 lineage^17^,^18^,^19^,^20^ (Supplementary Fig. 8). Factors that dictate reassortment efficiency between allelic influenza virus gene segments are poorly characterized^21^. Nevertheless, as internal genes like the *PB1* gene evolve slowly and do not show a high degree of host-specific evolution, they are frequently replaced during reassortment^22^. Thus, it remains possible that a novel pandemic virus may derive the PB1 segment from the North American ‘classical’ swine lineage or certain avian IAV populations encoding for PB1 398E resulting in completion of the ESIE-motif and potentially enhanced virulence.

### Restoration of the ESIE-motif increases virulence of pdmH1N1

**Figure 4:**
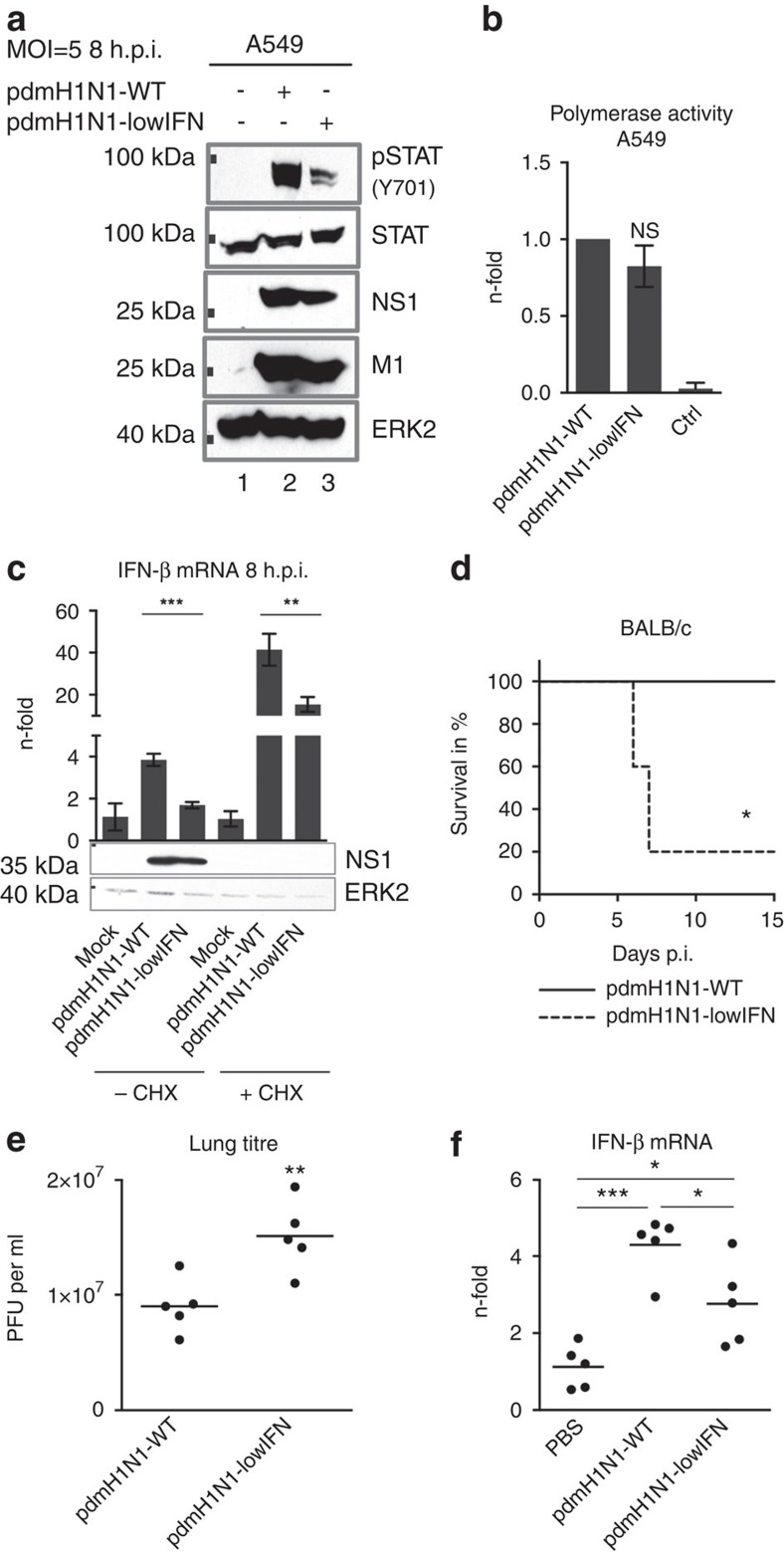
Introduction of the ESIE-motif in a pandemic H1N1 virus increases pathogenicity. (**a**) A549 cells were infected with the indicated viruses (multiplicity of infection (MOI)=5) and prepared for WB analysis 8 h.p.i. Enhanced type I IFN response following disruption of the ESIE-motif was analysed by probing for Y701-phosphorylation of STAT1 (full blot is shown in Supplementary Fig. 9a). Shown are representative results from three independent experiments. (**b**) Polymerase activity was measured in A549 cells. Cells not expressing PB1 served as a negative control (Ctrl); bars show mean±s.d. of three independent experiments. (**c**) A549 cells treated with cycloheximide (CHX) were infected with the indicated viruses (MOI=5) and prepared for qRT–PCR analysis. Elevated mRNA levels of *IFNβ* were measured following disruption of the ESIE-motif 8 h.p.i.; bars show mean±s.d. of three independent experiments. CHX treatment was controlled by WB analysis of NS1 (full blot is shown in Supplementary Fig. 9b). (**d**–**f**) BALB/c mice (*n*=5 per group) were infected i.n. with 10^4^ PFU and (**d**) examined daily for survival for 15 days p.i. (**e**) Virus titres of infected mouse lungs were determined 3 days p.i. (**f**) Elevated mRNA levels of *IFNβ* were measured 2 days p.i. Depicted are fold changes compared with PBS-inoculated mice. (**e**,**f**) Each dot represents an individual mouse; bars show mean. For statistically analysis (**b**,**c** and **f**), analysis of variance with Tukey’s test for multiple comparisons, (**d**) log-rank (*χ*^2^) test for statistical analysis of Kaplan–Meier survival data and (**e**) Student’s *t*-test were performed; *P<0.05, **P<0.01, ***P<0.001, NS=not statistically significant.

As an experimentally proof of this hypothesis, we introduced the functional ESIE-motif into the 2009 pandemic H1N1 virus, a strain usually lacking the complete motif by carrying PB1 398D. We observed enhanced antagonism of the type I IFN response (Fig. 4a, Supplementary Fig. 9a) independent of significant changes in polymerase activity (Fig. 4b) and even independent of viral replication (Fig. 4c). Increased morbidity and mortality were accompanied by enhanced viral load and diminished IFN-β mRNA levels in the lung of mice (Fig. 4d–f). This suggests the risk of multiple host lineage reassortant viruses—like foregone the 2009 pandemic H1N1 virus—with increased virulence mediated by the ESIE-motif.

Of note, a recombinant 2009 pandemic H1N1 virus mutant with PB1 398G—an amino acid change that disrupts the motif similar to the virus mutants H1N1-hIFN, H5N1-hIFN and H7N7-hIFN presented here—was highly attenuated (data not shown), suggesting the type I IFN antagonistic properties mediated by PB1 398D as sufficient for viral fitness, but PB1 398E as even more potent variant. In contrast to glycine (G), aspartic acid (D) and glutamic acid (E) both are negatively charged amino acids of related structure, suggesting a vital role of the position 398 in the PB1 protein.

In summary, we visualized IAV vRNPs in three-dimensional high-resolution light microscopy as helical-like structures associated with the mitochondrial outer membrane, despite the absence of an early type I IFN response. We identified PB1 and PA as new players in the type I IFN inhibitory strategy evolved by IAV, filling the gap in knowledge regarding early suppression of antiviral immune responses. The functional plasticity of IAV polymerase proteins emphasizes the evolutionary pressure for viruses with small genomes to evolve multifunctional proteins with temporally and spatially separated functions.

## Methods

### Viruses, cells, infection conditions and inhibitors

The following viruses were used: A/Puerto-Rico/8/34 (H1N1), A/Thailand/1(KAN-1)/2004 (H5N1), mouse-adapted A/Seal/Massachusetts/1/80 (H7N7)^23^ and the pandemic H1N1 strain A/Hamburg/04/2009 (pdmH1N1). Recombinant viruses were generated using the pHW2000-based reverse genetic system (a kind gift of Dr Robert G. Webster, Memphis, TN, USA)^24^. The following mutations were introduced by site-directed mutagenesis and confirmed by sequencing: H1N1-hIFN PB1 E398G PB1 S524G PB1 I563R PA E351K; H5N1-hIFN PB1 D398G PB1 S524G PA E351K; H7N7-hIFN PB1 D398G PB1 S524G PA E351K; pdmH1N1-lowIFN PB1 D398E PB1 R563I and H1N1-NS1 E96A NS1 E97A^8^. Primer sequences are provided in Supplementary Table 1. For generation of recombinant viruses, HEK293 were transfected with 1 μg of each of the eight plasmids using Trans-IT LT1 (Mirus) according to the manufacturer’s protocol. Cells were cultivated in DMEM supplemented with 0.2% bovine serum albumin. 6 h post transfection, medium was changed to DMEM supplemented with 0.2% bovine serum albumin, 1 mM MgCl_2_, 0.9 mM CaCl_2_, 100 U ml^−1^ penicillin, 0.1 mg ml^−1^ streptomycin and 0.3 μg ml^−1^ TPCK-trypsin. 48 h post transfection, the supernatant was used for infection of Madin–Darby canine kidney cells (MDCK). All viruses were propagated in MDCK cells and handled under biosafety level 2 or 3 conditions. Virus titres were determined by plaque assay and haemagglutinin titration assay^25^.

The human lung epithelial cell line A549 (American Type Culture Collection (ATCC)) was cultivated in DMEM (Lonza), whereas MDCK cells (ATCC) were cultivated in MEM (Lonza), both supplemented with 10% heat-inactivated fetal calf serum. NHBE cells (Lonza) were cultivated following the manufacturer’s protocol. U937 (ATCC) were cultivated in RPMI media (Lonza) supplemented with 10% fetal calf serum upon differentiation with 200 nM phorbol-12-myristate-13-actete (Sigma).

For infection, cells were inoculated with IAV at the indicated multiplicities of infection in PBS supplemented with 0.2% bovine serum albumin, 1 mM MgCl_2_, 0.9 mM CaCl_2_, 100 U ml^−1^ penicillin and 0.1 mg ml^−1^ streptomycin for 30 min at 37 °C following incubation with either DMEM or MEM supplemented with 0.2% BSA, 100 U ml^−1^ penicillin and 0.1 mg ml^−1^ streptomycin. Infections were performed in technical duplicates (western blot analysis) or triplicates (quantitative real-time PCR analysis).

Cycloheximide (Sigma) was used at 50 μg ml^−1^ and actinomycin D (Sigma) at 1 μg ml^−1^. Both were added to the medium 30 min before infection and throughout the infection period.

### Minireplicon luciferase assay and transient transfection

Cells were transfected with Trans-IT LT1 (Mirus) according to the manufacturer’s protocol. To generate pcDNA3-H1N1-PB1-WT, pcDNA3-H1N1-PA-WT, pcDNA3-H1N1-PB1-hIFN and pcDNA3-H1N1-PA-hIFN, H1N1-WT- and H1N1-hIFN-derived PB1 and PA open reading frames were amplified from pHW2000-H1N1-PB1 or pHW2000-H1N1-PA and linked to a *Not*I and *Xba*I restriction site using the primer sets 5NotPB1 (5′-ATAAGAATGCGGCCGCTATGGATGTCAATCCG-3′) and 3XbaPB1 (5´-GTGTCTAGACTATTTTTGCCGTCTGAGCTCT-3′), and 5NotPA (5′-ATAAGAATGCGGCCGCTATGGAAGATTTTGTG-3′) and 3XbaPA (5′-GTGTCTAGACTAACTCAATGCATGTGTAAGG-3′) and inserted into pcDNA3. The generated plasmids together with pcAGGS-RIG-I-FLAG^26^ were used for overexpression of the corresponding proteins and empty vectors served as control.

For the minireplicon luciferase assay, cells were transfected with plasmids encoding an antisense luciferase reporter gene flanked by the influenza promoters of the M-segment (pHW72-luc-vector; 0.5 μg) and the polymerase subunits PB2, PB1, PA (0.25 μg each) and NP (0.5 μg) of A/Puerto-Rico/8/34 (H1N1; pHW2000-vector) or A/Hamburg/04/2009 (pdmH1N1; pHW2000-vector). As negative control, cells were transfected with the same set of plasmids but leaving out the PB1 expression construct. 24 h post transfection, cells were harvested and measurement of luminescence was performed as described earlier^27^.

### Microscopy

A549 cells were grown on #1.5 chambered cover glasses. 3 h.p.i., cells were fixed for 10 min with 4% (v/v) paraformaldehyde and 0.1% (v/v) glutaraldehyde in PBS. Cells were briefly washed before quenching of reactive groups with 0.1% (w/v) sodium borohydride for 10 min and rinsed with TBS, before permeabilization with TBS containing 0.1% (v/v) Triton X-100 for 3 min. Cells were rinsed with TBS and blocked with TBS containing 2% bovine serum albumin before labelling of vRNPs with antibodies specific for viral NP (1:1,000; AbD serotec; MCA400), the outer mitochondrial membrane (1:1,000; Tom20; SCBT; sc-11415), RIG-I (1:500; Cell Signaling; #4520) and MAVS (1:200; Pierce; PA5-17256). Nuclear stain was performed using Hoechst 33258 (Life Technologies; H3569). Following incubation, cells were rinsed with TBS, and primary antibodies were detected using goat anti-mouse AF568 (1:2,000; Life Technologies; A11031), goat anti-rabbit AF647 (1:2,000; Life Technologies; A21245) goat anti-mouse ATTO488 (1:2,000; Labome; 610152121) secondary antibodies. Biotinylation was performed using EZ-Link Sulfo-NHS-LC-Biotin and Biotinylation Kits according to the manufacturer’s protocol (Pierce; 21435). Three-dimensional spinning-disc confocal images were acquired with a step size of 0.4 μm, and analysed using Imaris 7.6.5 software (Bitplane). Surfaces were created to segment mitochondria and vRNPs. The distance from each vRNP to the closest mitochondrial surface was measured via an intensity to distance transform. At least seven images were analysed per experiment (*n*=3) with at least 40 cells analysed per group.

Samples were similarly prepared for analysis of viral NP, RIG-I, MAVS and mitochondrial proteins using STORM^28^. In brief, samples prepared as above were imaged using the N-STORM system built on a Nikon-Ti-E inverted microscope with a CFI SR Apo 100X TIRF objective having an numerical aperture of 1.49. Illumination was provided using an Agilent laser launch containing four laser lines, which were combined into a single optical fiber and collimated onto the back focal plane of the objective. Nominal output was measured as 20 mW for 405 nm, 80 mW for 488 and 561 nm, and 125 mW for 647 nm. Fluorescence emission was collected using an iXon DU897 Ultra EMCCD camera running at frame rate of 109 f.p.s. with EM Gain 17 mHz at 16-bit. 3D STORM was accomplished by insertion of an astigmatic lens into the light path before detection by a high-speed camera^29^. Calibration of z data was performed using 100 nm fluorescent beads and a z-stepping range of 800 nm (ref. 29). To facilitate STORM using AF568 and AF647 fluorescent probes, samples were imaged for 40,000 frames in buffer comprised of 50 mM Tris-HCl, pH 7.0, 50 mM NaCl and 10% glucose, and containing freshly prepared 10 mM cysteamine, 0.2 mg ml^−1^ catalase and 0.5 mg ml^−1^ glucose oxidase. The reconstructed 3D STORM image was created using algorithms for molecule identification and drift correction as previously described^29^ and employed by Nikon Instruments.

### Electrophoresis, western blotting and IP

After infection, cells were lysed on ice with RIPA lysis buffer (25 mM Tris-HCl, pH 8.0, 137 mM NaCl, 10% glycerol, 0.1% SDS, 0.5% deoxycholic acid (DOC), 1% NP40, 2 mM EDTA pH 8.0, 5 μg ml^−1^ leupeptin, 5 μg ml^−1^ aprotinin, 0.2 mM Pefabloc, 1 mM sodium vanadate and 5 mM benzamidine) for 30 min and cleared from debris by centrifugation at 14,000 r.p.m. for 10 min at 4 °C. Protein concentrations were determined by Bradford assay. For SDS–polyacrylamide gel electrophoresis (PAGE) samples were prepared with 5 × denaturing sample buffer (312 mM Tris-HCl, pH 6.8, 10% SDS, 50% glycerol, 25% β-mercaptoethanol and 0.01% bromphenol blue), heated for 10 min at 95 °C, loaded on a SDS–polyacrylamide gel with a 4% stacking over a 10% resolving gel and separated at 15 mA per gel using electrophoresis buffer (25 mM Tris, 250 mM glycine, 0.1% SDS). Proteins were transferred to a nitrocellulose membrane using a wet blotting system at 400 mA for 60 min in transfer buffer (25 mM Tris, 192 mM glycin, 20% methanol). Blocking and probing with antibodies were performed following manufacturer’s protocols. STAT1 phosphorylation was analysed with a phospho-specific anti-STAT1 [pY701] rabbit mAb (1:1,000; Cell Signaling; #9171). Viral proteins were detected in western blot (WB) analysis by using an anti-NS1 mouse mAb (1:500; clone NS1-23-1; developed at the IMV, Muenster, Germany) and anti-M1 mouse mAb (1:1,000; Serotec; MCA401). Anti-ERK2 (C-14) rabbit pAb (1:1,000; Santa Cruz; sc-154) and anti-STAT1 rabbit pAb (1:1,000; Cell signaling; #9172) were used in WB analysis for loading controls. The oligomeric active state of IRF3 was analysed in native PAGE^30^ followed by WB probing for IRF3 with an anti-IRF3 rabbit mAb (1:1000, Santa Cruz; sc-9082). Protein bands were visualized by standard enhanced chemiluminescence reaction. WBs were quantified using ImageJ 1.48v. pSTAT1 bands were normalized to STAT1 bands. NS1 and M1 bands were normalized to ERK2 bands. Depicted are the fold changes of band intensities of mutant viruses compared with wild-type viruses.

For IP cells were processed as described elsewhere^31^. Briefly, cells were lysed on ice with triton lysis buffer (20 mM Tris-HCl, pH 7.4, 137 mM NaCl, 10% glycerol, 1% Triton X-100, 2 mM EDTA, 50 mM sodium glycerophosphate, 20 mM sodium pyrophosphate, 5 μg ml^−1^ leupeptin, 5 μg ml^−1^ aprotinin, 0.2 mM Pefabloc, 1 mM sodium vanadate and 5 mM benzamidine) for 30 min and cleared by centrifugation at 14,000 r.p.m. for 10 min at 4 °C following incubation with protein A agarose for 1 h in an overhead shaker to minimize unspecific precipitation. Anti-RIG-I rabbit mAb (2 μg; Cell Signaling; #3743) was coupled to protein A agarose by incubation at 300 r.p.m. for 3 h at 4 °C before samples were added and incubated in an overhead shaker over night at 4 °C. Samples were washed three times with triton lysis buffer, prepared with 5 × denaturating sample buffer, heated for 10 min at 95 °C and subjected to SDS–PAGE. As IP-input controls, lysates were directly subjected to SDS–PAGE and WB analysis. RIG-I was visualized by the RIG-I antibody mentioned above. PB1 was detected by anti-PB1 rabbit Ab (1:1,000; GenScript; A01467). Anti-Tubulin rabbit mAb (1:1,000; Cell Signaling; #2128) was used for loading controls.

### Quantitative real-time PCR (qRT–PCR)

Total RNA was isolated with the RNeasy mini kit (Qiagen) according to the manufacturer’s protocol. For cDNA synthesis, a total of 1 μg RNA and M-MLV Reverse Transciptase (Promega) were used. PCR was performed with QuantiFast SYBR Green PCR Kit (Qiagen) and carried out on a 7300 Real-Time PCR System (Applied Biosystem). Human IFN-β mRNA was detected with a specific QuantiTect primer assay (Qiagen; Hs_IFNB1_1_SG) and normalized against HPRT (Qiagen; Hs_HPRT_1_1_SG) using the 2^−ΔΔCT^ method^32^. Upregulation is depicted in relation to non-infected cells. Transcriptome analysis for type I IFN-related genes in U937 cells was performed by using the RT^2^ Profiler PCR Array Human Interferons and Receptors (Qiagen, PAHS-064) according to the manufacturer’s instructions. Upregulation in PR8-hIFN-infected cells is depicted in relation to PR8-WT-infected cells.

### Ethics statement

The Animal Care and Use Committee at St. Jude Children’s Research Hospital approved all mouse studies with wild-type mice. Experiments with IFN receptor-deficient mice were approved by the local animal welfare authority (Regierungspräsidium Freiburg) according to the local law and animal ethics regulations.

### Mouse experiments

Eight-week-old female BALB/c, C57Bl/6, DBA/J2, *Stat1*^*−/−*^ mice (Jackson Laboratory) and *Ifnar1*^*−/−*^*Ifnlr1*^*−/−*^ mice (male and female in mixed groups)^33^ were used for the mouse studies. For infection, mice were anaesthetized either by intraperitoneal injection of 100 mg ketamine (Ceva) and 5 mg of xylazine (Ceva) per kg of body weight or by inhalation of 2.5% isoflurane (Baxter Healthcare Corporation). Mice were infected intra nasally (i.n.) with the indicated viruses and plaque-forming units (PFUs) in a 50 μl volume and monitored daily for disease symptoms, body weight and survival. Owing to animal welfare restrictions, mice were euthanatized upon a body weight loss of 25%.

### Virus titres and cytokine levels in mouse lungs

For quantification of infectious virus particles in infected mouse lungs, mice were euthanatized on day 3 p.i. and lungs were collected in PBS. The lungs were homogenized using a FastPrep-24 homogenisator with Lysing Matrix D (MP Biomedical). PBS was added to obtain 10% tissue homogenates. The samples were centrifuged and the supernatants were used for determination of virus titres in standard plaque titration as described elsewhere^24^. For analysing cytokine levels in total mouse lungs, animals were euthanatized on day 2 p.i. and lungs were collected in Trizol. For isolation of RNA, a modification of the RNAzol method was used^34^. Reverse transcription and qRT–PCR reactions were conducted as described above. Murine IFN-β mRNA was detected with a specific QuantiTect primer assay (Qiagen; ifb1_1_SG mouse) and normalized against GAPDH (Qiagen; gapdh_3_SG mouse).

### Phylogenetic analysis

All complete avian, human and swine PB1-coding sequences were acquired from FLUDB and translated into proteins. They were clustered at 98% identity to remove redundancy using USEARCH and aligned using MUSCLE with default options^35^,^36^. Phylogenetic tree construction was performed using BEAST with the FLU substitution model and allowing gamma-distributed rates across sites^37^,^38^. A relaxed molecular clock was assumed, with log-normally distributed branch rates. Default priors were used in all cases except for ucld.mean (exponential with mean 0.005) and ucld.stdev (exponential with mean 0.333). Fifty million generations of MCMC were performed. Phylogenetic analysis of representative viruses of the North American swine lineage and Eurasian swine lineage was conducted using MEGA5 (ref. 39).

## Author contributions

Sw.L. designed the study, collected and analysed data and wrote the paper; E.R.H. collected data and was involved in study design and manuscript preparation; C.G. performed spinning disc microcopy and STORM analysis; D.A., R.D. and P.S. collected data; G.W. and R.C. performed bioinformatical sequence analysis; D.R.G. and T.W. were involved in study design, J.A.M., St.L. and C.E. were involved in study design and manuscript preparation.

## Additional information

**How to cite this article:** Liedmann, S. *et al.* Viral suppressors of the RIG-I-mediated interferon response are pre-packaged in influenza virions. *Nat. Commun.* 5:5645 doi: 10.1038/ncomms6645 (2014).

## Supplementary Material

Supplementary Information Supplementary Figures 1-9, Supplementary Table 1 and Supplementary References

Video - Supplementary Movie1 High-resolution visualization of internalized IAV vRNPs revealed helical-like structure.
