# Supplementary material for: Viral suppressors of the RIG-I-mediated interferon response are pre-packaged in influenza virions
Source: Nat Commun. 2014 Dec 9;5:5645. doi: 10.1038/ncomms6645 (PMC4268707; doi:10.1038/ncomms6645)
Supplement: Supplementary Information — Supplementary Figures 1-9, Supplementary Table 1 and Supplementary References [file ncomms6645-s1.pdf]

SUPPLEMENTARY INFORMATION

Viral suppressors of the RIG-I-mediated interferon response are pre-packaged in influenza virions

Authors: Swantje Liedmann[1], Eike R. Hrincius[2], Cliff Guy[3], Darisuren Anhlan[1], Rüdiger Dierkes[1], Robert Carter[4], Gang Wu[4], Peter Staeheli[5], Douglas R. Green[3], Thorsten Wolff[6], Jonathan A. McCullers[2,7], Stephan Ludwig[1,8]* and Christina Ehrhardt[1,8]

Supplementary Figure 1

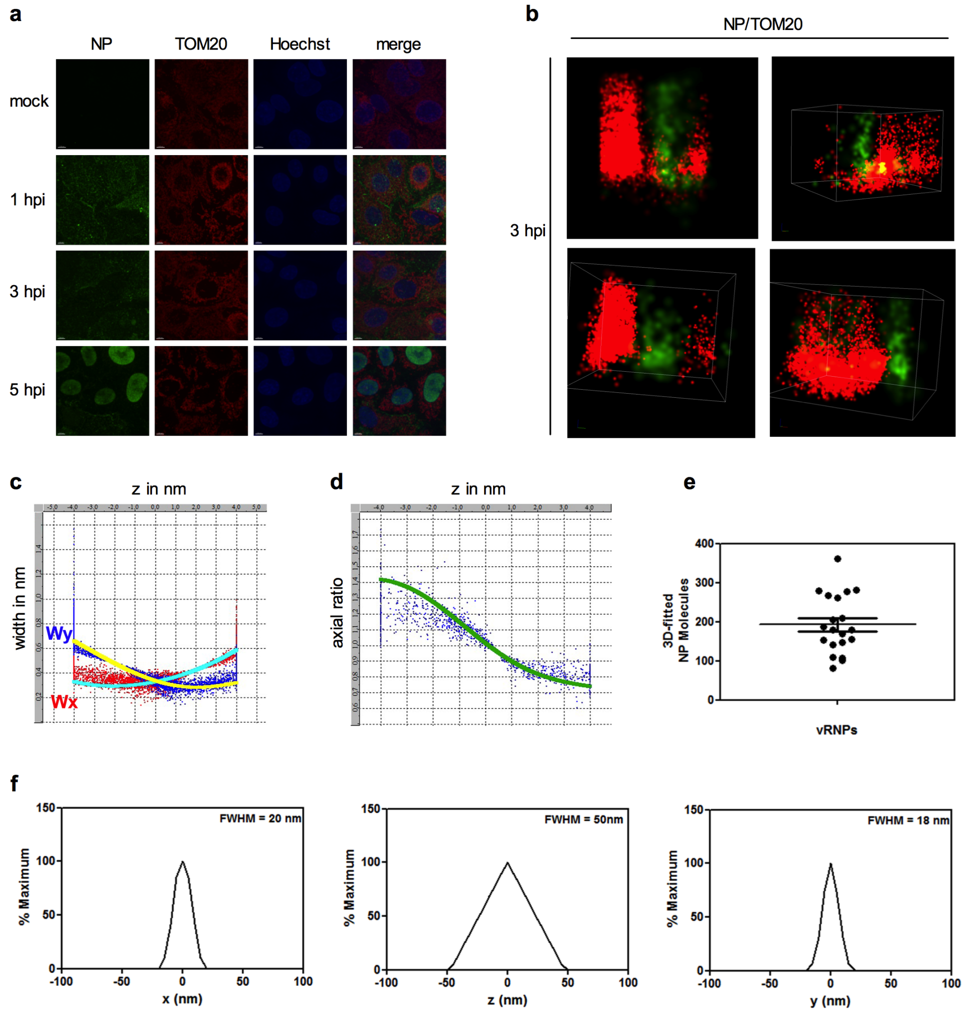

Supplementary Figure 1: Determination of three-dimensional localization accuracy.

A549 cells were infected with H1N1-WT (MOI = 50) and prepared for immunofluorescence analysis. (a) Spinning disk laser scanning confocal microscopy revealed cytosolic vRNPs (green: NP-AF568) in the mitochondrial network (TOM20-AF647) previous to import into the nucleus (Hoechst) at 3 hpi. Nuclear import was completed at 5 hpi. Scale bars represent 5 µm. Shown are representative images from three independent experiments. (b) Three-dimensional stochastic optical reconstruction microscopy (STORM) revealed vRNPs (green: NP-AF568) as helical-like structure next to the outer mitochondrial membrane (red: TOM20-AF647) 3 hpi. (c, d and f) Three-dimensional localization accuracy of astigmatic STORM imaging was determined using fluorescent microbeads. (c) Width of the localized molecules (Wx or Wy) and (d) the axial ratio of the molecules are plotted against the z stepping range. (e) The number of molecules fitted to the three-dimensional calibration curves are shown for 20 randomly selected vRNPs. (f) The spatial distributions of the localizations within the bead molecules are presented for the x, y and z dimensions, with respective FWHM values as noted.

Supplementary Figure 2

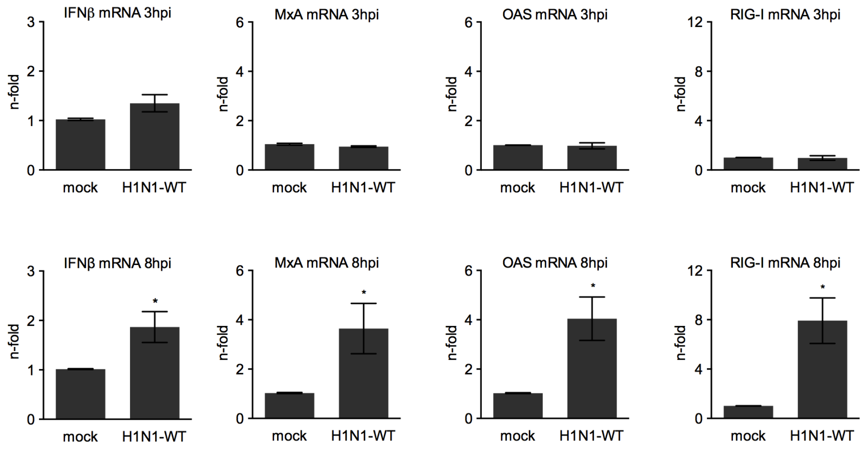

Supplementary Figure 2: The type I IFN signaling pathway is not activated at 3 hours post infection.

A549 cells were infected with H1N1-WT (MOI = 5) and prepared for qRT-PCR-analysis. MessengerRNA levels of *IFNβ, RIG-I*, *MxA* and *OAS* were unchanged at 3 hpi. Elevated mRNA levels were measured at 8 hpi; bars show mean ± SD of three independent experiments. For statistically analysis Students t-test was performed; *P<0.05.

Supplementary Figure 3

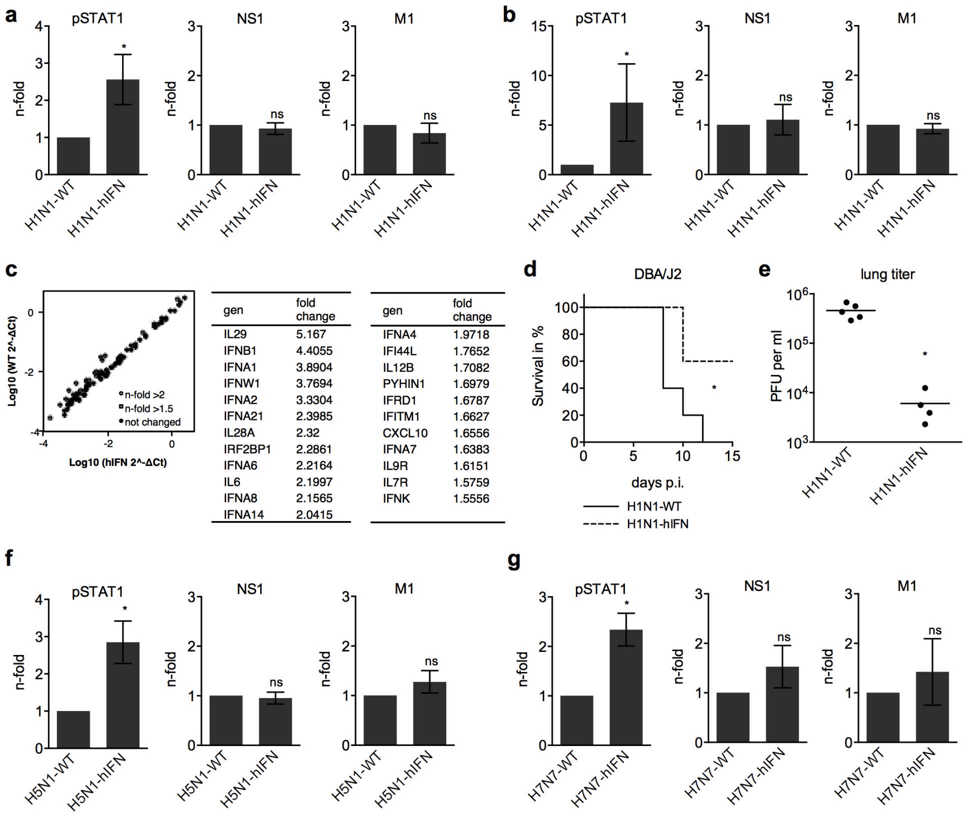

gen	fold change
IL29	5.167
IFNB1	4.4055
IFNA1	3.8904
IFNW1	3.7694
IFNA2	3.3304
IFNA21	2.3985
IL28A	2.32
IRF2BP1	2.2861
IFNA6	2.2164
IL6	2.1997
IFNA8	2.1565
IFNA14	2.0415

gen	fold change
IFNA4	1.9718
IFI44L	1.7652
IL12B	1.7082
PYHIN1	1.6979
IFRD1	1.6787
IFITM1	1.6627
CXCL10	1.6556
IFNA7	1.6383
IL9R	1.6151
IL7R	1.5759
IFNK	1.5556

Supplementary Figure 3: Quantification of Western Blots, expression analysis of IFN-related genes and pathogenicity in DBA/J2 mice.

Western Blots were quantified using IMAGEJ 1.48v. Representative WB are presented in (a, b) Figure 2a and (f, g) Figure 2k. pSTAT1 bands were normalized to STAT1 bands. NS1 and M1 bands were normalized to ERK2 bands. Depicted are fold changes of mutant viruses compared to wild type viruses; quantified were WB of three independent experiments, bars show mean ± SD. (c) U937 cells were infected with H1N1-WT and H1N1-hIFN (MOI = 5) and prepared for qRT-PCR-analysis. Enhanced (>1.5-fold) expression of type I IFN-related genes following disruption of the ESIE-motif was analyzed in an 88-gene-transcriptome analysis 8 hpi. (d, e) DBA/J2 mice (n = 5 per group) were infected i.n. with 10 PFU of the indicated viruses. (d) Mice were examined daily for survival for 15 days p.i. (e) Virus titers of

infected mouse lungs were determined 3 days p.i. Each dot represents an individual mouse; bars show mean. For statistically analysis (a, b, e-g) Students t-test and (d) log-rank (chi-square) test for statistical analysis of Kaplan-Meier survival data were performed; *P<0.05, ns = not statistically significant.

Supplementary Figure 4

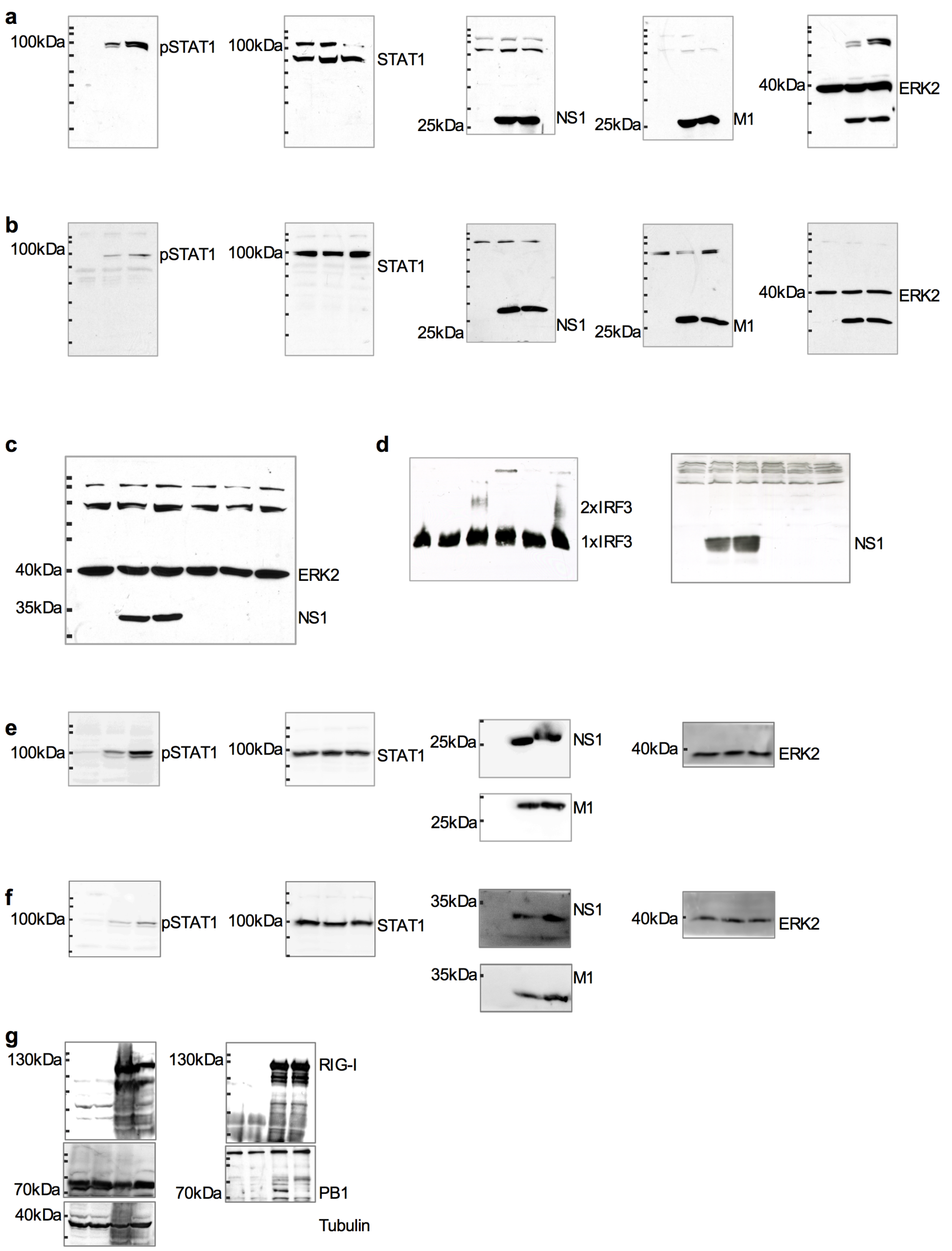

Supplementary Figure 4: Original Western Blots.

Shown are the original Western Blots from (a-f) Figure 2 and (g) Figure 3.

Supplementary Figure 5

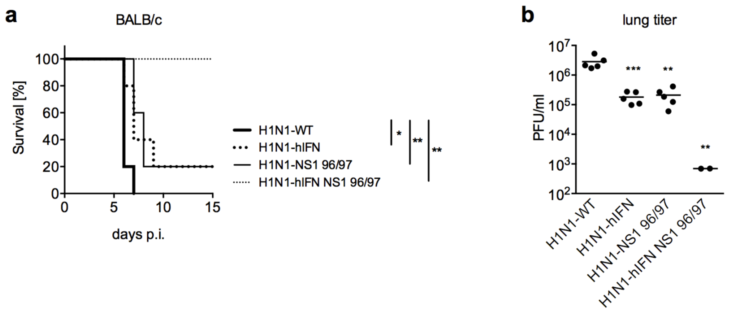

Supplementary Figure 5: IFN antagonism of PB1/PA impacts virulence regulation similar to RIG-I-mediated IFN antagonism of NS1.

(a, b) BALB/c mice (n = 5 per group) were infected i.n. with 10^4 PFU of the indicated viruses. (a) Mice were examined daily for survival for 15 days p.i. (b) Virus titers of infected mouse lungs were determined 3 days p.i. Each dot represents an individual mouse; bars show mean. For statistically analysis (a) log-rank (chi-square) test for statistical analysis of Kaplan-Meier survival data and (f) ANOVA with Tukey test for multiple comparisons were performed *P<0.05, **P<0.01, ***P<0.001.

Supplementary Figure 6

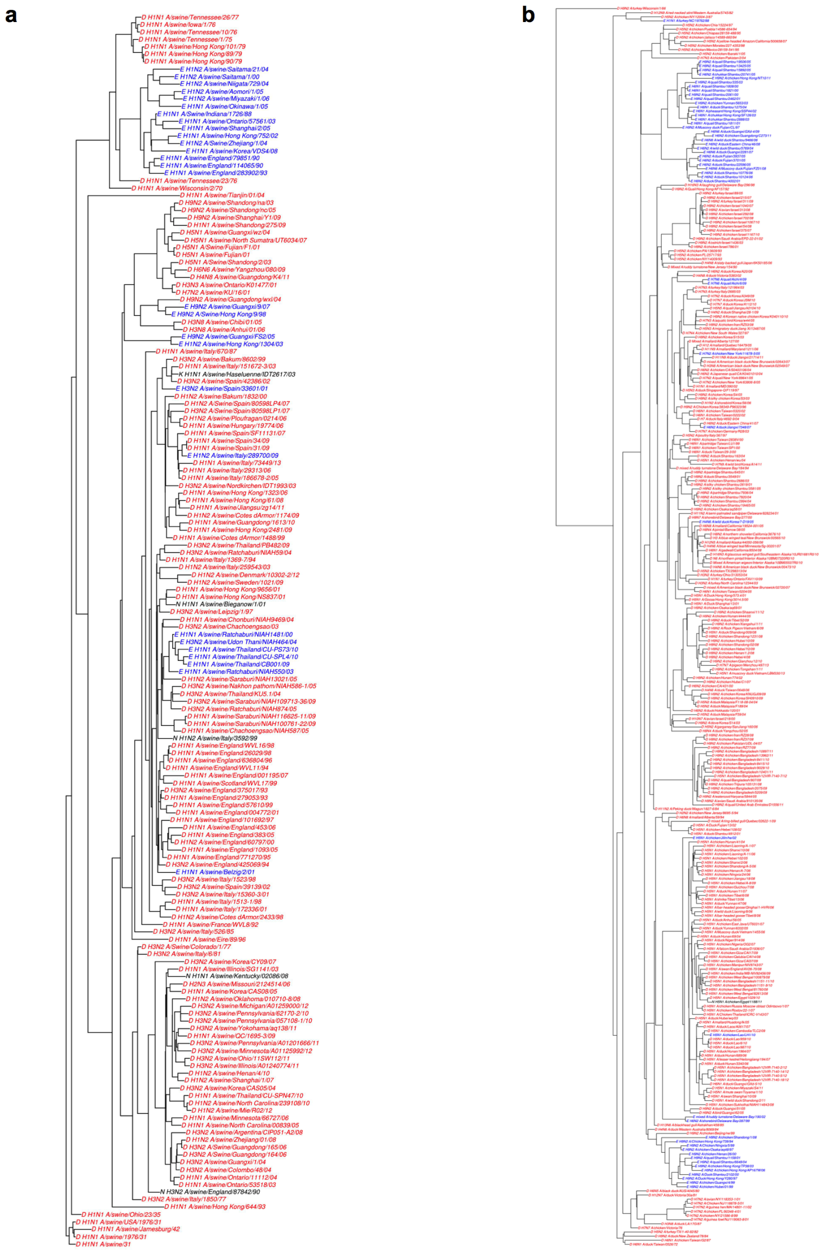

Supplementary Figure 6: Evolutionary dynamics of the ESIE-motif in swine and avian IAV.

PB1 protein sequences of (a) swine and (b) avian IAV were used to construct phylogenetic tree with BEAST. Red color marks IAV with PB1 398D; blue color marks IAV with PB1 398E.

Supplementary Figure 7

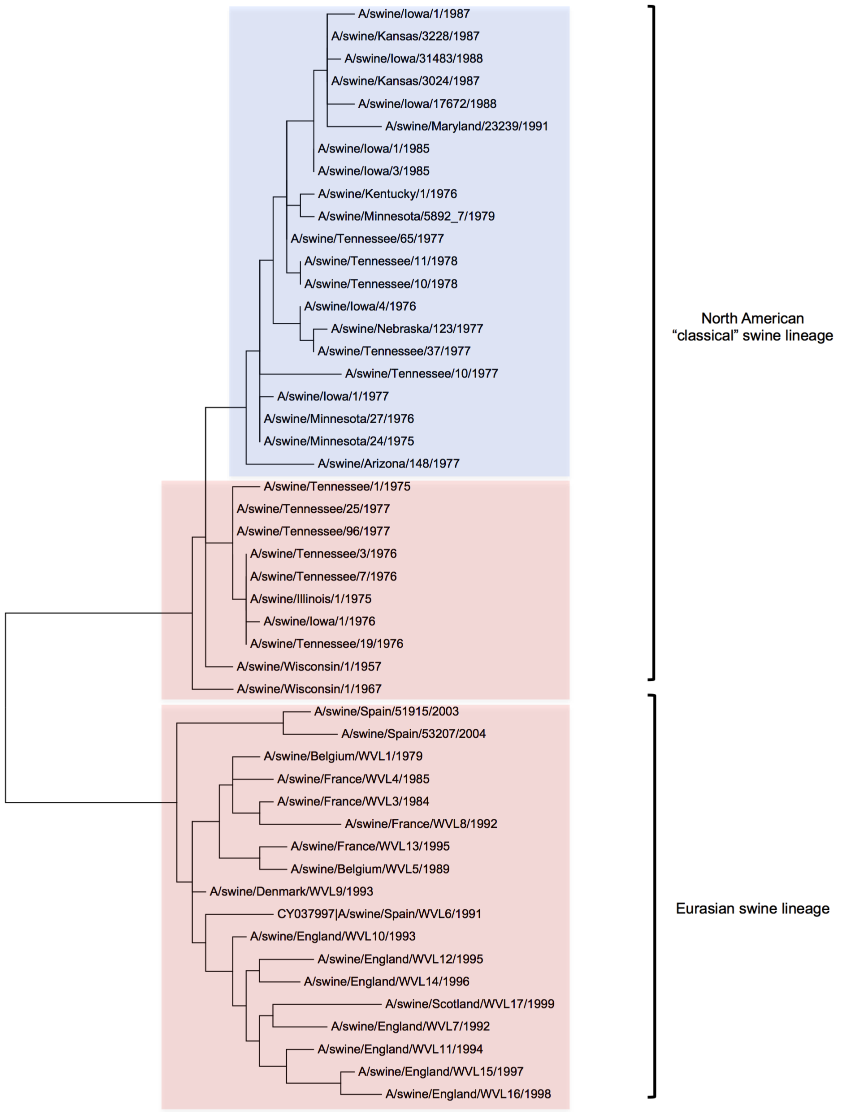

Supplementary Figure 7: Completion of the ESIE-motif in the North American swine lineage and evolution of swine IAV, 1918-2009.

Amino acid sequences of the PB1 genes of representative IAV of the North American "classical" swine lineage and the Eurasian swine lineage were aligned by MUSCLE; the phylogenetic tree was constructed by using MEGA5. Red background marks IAV with PB1 398D, blue background marks IAV with PB1 398E.

Supplementary Figure 8

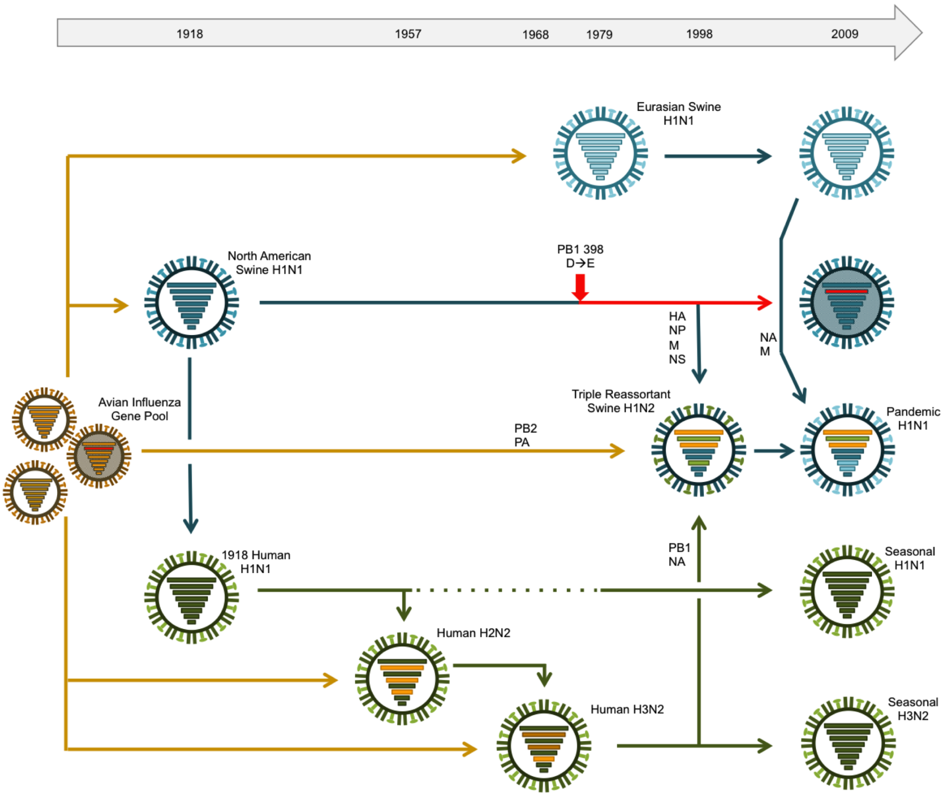

Supplementary Figure 8: Evolution of swine IAV, 1918-2009.

Yellow arrows indicate derivation of gene segments from the avian IAV gene pool; evolutionary paths of swine IAV are shown with blue arrows, of human IAV with green arrows. The red arrow marks the D to E change at position 398 in the PB1 protein within the North American swine lineage. Today PB1 398E is present in an avian IAV population and persists in the North American swine lineage (shadowed virus particles with red gene segment). Figure is modified from.[1, 2]

Supplementary Figure 9

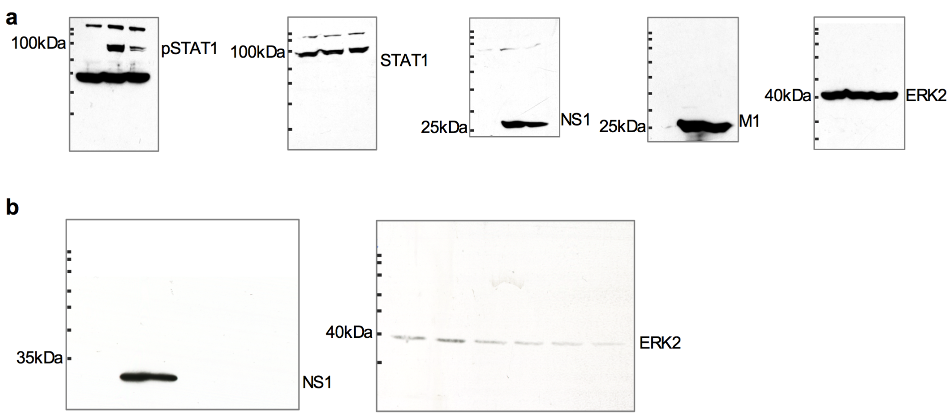

Supplementary Figure 9: Original Western Blots.

Shown are the original Western Blots from Figure 4.

SUPPLEMENTARY TABLE

Supplementary Table 1: Mutagenesis primer sequences

H1N1-hIFN PB1 E398G for	CCGCTCTTAATAGGGGGGACTGCATC
H1N1-hIFN PB1 E398G rev	GATGCAGTCCCCCCTATTAAGAGCGG
H1N1-hIFN PB1 S524G for	GAGTCAGCGGACATGGGTATTGGAGTTACTG
H1N1-hIFN PB1 S524G rev	CAGTAACTCCAATACCCATGTCCGCTGACTC
H1N1-hIFN PB1 I563R for	CGTACCGATGCCATAGAGGTGACACACAAATAC
H1N1-hIFN PB1 I563R rev	GTATTTGTGTGTCACCTCTATGGCATCGGTACG
H1N1-hIFN PA E351K for	GGACATTGAGAATAAGGAGAAAATTCCAAAG
H1N1-hIFN PA E351K rev	CTTTGGAATTTTCTCCTTATTCTCAATGTCC
H5N1-hIFN PB1 D398G for	TCTATTAATAGGTGGTACAGCC
H5N1-hIFN PB1 D398G rev	GGCTGTACCACCTATTAATAGA
H5N1-hIFN PB1 S524G for	GCCGACATGGGCATTGGTGTT
H5N1-hIFN PB1 S524G rev	AACACCAATGCCCATGTCGGC
H5N1-hIFN PA E351K for	ATTGAAAATAAGGAGAAAATC
H5N1-hIFN PA E351K rev	GATTTTCTCCTTATTTTCAAT
H7N7-hIFN PB1 D398G for	CTTCTAATAGGTGGTACAGCC
H7N7-hIFN PB1 D398G rev	GGCTGTACCACCTATTAGAAG
H7N7-hIFN PB1 S524G for	GCTGACATGGGCATTGGAGTA
H7N7-hIFN PB1 S524G rev	TACTCCAATGCCCATGTCAGC
H7N7-hIFN PA E351K for	ATTGAAAATAAAGAGAAGATT
H7N7-hIFN PA E351K for	AATCTTCTCTTTATTTCAAT
pdmH1N1-lowIFN PB1 D398E for	CTTCTAATAGAGGGCACAGCA
pdmH1N1-lowIFN PB1 D398E rev	TGCTGTGCCCTCTATTAGAAG
pdmH1N1-lowIFN PB1 R563I for A	AGGTGCCATAGAGGAGACACA
pdmH1N1-lowIFN PB1 R563I rev A	TGTGTCTCCTCTATGGCACCT
pdmH1N1-lowIFN PB1 R563I for B	AGGTGCCATATAGGAGACACA
pdmH1N1-lowIFN PB1 R563I rev B	TGTGTCTCCTATATGGCACCT
H1N1 NS1 E96A for	CATGACTCTTGCGGAAATGTCAAGG
H1N1 NS1 E96A rev	CCTTGACATTTCCGCAAGAGTCATG
H1N1 NS1 E97A for	CATGACTCTTGCGGCAATGTCAAGG
H1N1 NS1 E97A rev	CCTTGACATTGCCGCAAGAGTCATG

SUPPLEMENTARY REFERENCES

1. Morens, D. M., Taubenberger, J. K., Fauci, A. S. The persistent legacy of the 1918 influenza virus. *N. Engl. J. Med.* **361,** 225-229 (2009).
2. Taubenberger, J. K., Kash, J. C. Influenza virus evolution, host adaptation, and pandemic formation. *Cell Host Microbe* **7,** 440-451 (2010).
